# Prediction and imitation in speech

**DOI:** 10.3389/fpsyg.2013.00340

**Published:** 2013-06-21

**Authors:** Chiara Gambi, Martin J. Pickering

**Affiliations:** Department of Psychology, University of EdinburghEdinburgh, UK

**Keywords:** imitation, convergence, forward models, simulation, prediction error

## Abstract

It has been suggested that intra- and inter-speaker variability in speech are correlated. Interlocutors have been shown to converge on various phonetic dimensions. In addition, speakers imitate the phonetic properties of voices they are exposed to in shadowing, repetition, and even passive listening tasks. We review three theoretical accounts of speech imitation and convergence phenomena: (i) the Episodic Theory (ET) of speech perception and production (Goldinger, 1998); (ii) the Motor Theory (MT) of speech perception (Liberman and Whalen, 2000; Galantucci et al., 2006); (iii) Communication Accommodation Theory (CAT; Giles and Coupland, 1991; Giles et al., 1991). We argue that no account is able to explain all the available evidence. In particular, there is a need to integrate low-level, mechanistic accounts (like ET and MT), and higher-level accounts (like CAT). We propose that this is possible within the framework of an integrated theory of production and comprehension (Pickering and Garrod, 2013). Similarly to both ET and MT, this theory assumes parity between production and perception. Uniquely, however, it posits that listeners simulate speakers' utterances by computing forward-model predictions at many different levels, which are then compared to the incoming phonetic input. In our account phonetic imitation can be achieved via the same mechanism that is responsible for sensorimotor adaptation; i.e., the correction of prediction errors. In addition, the model assumes that the degree to which sensory prediction errors lead to motor adjustments is context-dependent. The notion of context subsumes both the preceding linguistic input and non-linguistic attributes of the situation (e.g., the speaker's and listener's social identities, their conversational roles, the listener's intention to imitate).

## Introduction

The communicative power of speech stems from the balance between systematicity and variability. Speech obeys rules set by each community of speakers, but it is also highly idiosyncratic, because of individual differences in factors such as linguistic experience (e.g., Bradlow et al., 1997) and vocal tract characteristics (Simpson, 2001). The present article is concerned with aspects of speech which are not entirely constrained by the grammar of the language the speaker uses or deterministically specified by her physical characteristics. However, such variation is sufficiently limited that it does not alter the message significantly. It therefore makes sense to ask whether such variation is affected by an individual's recent experience; that is, by the speech input she has just been exposed to.

There is evidence that comprehenders imitate the input they have been exposed to at many different levels, such as the lexical and the syntactic (Pickering and Garrod, 2004). Here, we focus our attention on imitation at the phonetic level. Listeners have been shown to imitate subtle phonetic variation in a speaker's voice. They do so unintentionally and under laboratory conditions, but also in more naturalistic conversational contexts. At least three different theoretical approaches have been proposed to account for a wide range of findings that have not always been consistent across studies and across experimental paradigms. These theoretical approaches are: (i) the Episodic Theory (ET) of speech perception and production (Goldinger, 1998); (ii) the Motor Theory (MT) of speech perception (Liberman and Whalen, 2000); (iii) Communication Accommodation Theory (CAT; Giles and Coupland, 1991; Giles et al., 1991).

In the following we present each theory and briefly review supporting evidence. Then we introduce a different theoretical approach, which we term the Simulation Theory (ST) of speech perception. This theory posits that listeners simulate speech input internally using speech production mechanisms, most importantly forward models (Guenther et al., 2006; Pickering and Garrod, 2007, 2013; Adank et al., 2010). We will then argue that the existing literature is compatible with this proposal. Finally, we will present some testable predictions of the theory.

In doing so, we hope to provide an account of phonetic imitation and convergence that is mechanistic in the sense that it specifies the nature of the cognitive mechanism that is responsible for this phenomenon. At the same time, the account should be able to explain why the extent of imitation is influenced by a variety of factors, particularly by social factors pertaining to the relationship between listener and speaker (see Section “Communication Accommodation Theory” below). Our aim, specifically, is to formulate hypotheses as to how such factors could modulate the cognitive mechanism underlying phonetic imitation.

## The episodic theory of speech perception and production

Goldinger (1998) introduced an experimental paradigm that has been later adopted by many phonetic imitation studies. Two sets of participants are involved: a set of speakers and a set of listeners. Speakers listen to utterances produced by a model and receive instructions to repeat them (either immediately, or with some delay). Listeners are presented with triplets of auditory stimuli in a so-called AXB task. The medially presented stimulus (X) is always an utterance produced by the model, while A and B are tokens of the same utterance (i.e., the same word) produced by the same speaker either as a repetition of the model utterance or at baseline (i.e., before exposure to the model, normally elicited in a reading task).

Goldinger (1998) found that listeners judge repeated tokens to be more similar to the model than baseline tokens. Listeners' judgments were taken as evidence that speakers spontaneously imitated the model's speech. Imitation was interpreted by Goldinger as supporting the ET with respect to the organization of the mental lexicon. ET posits that each individual percept (e.g., each heard word) leaves a trace in memory, and that such traces (echoes) contain detailed phonetic information, including specific characteristics of a speaker's voice. Each recent perceptual event can thus influence the mental representation of a word and subsequently affect production of the same word, given the assumption that the mental lexicon is shared between comprehension (i.e., perception) and production.

Other findings can be explained in terms of properties of memory traces. First, imitation is stronger when the delay between perception and production is shorter (Goldinger, 1998; Kappes et al., 2009). This is expected because echoes “fade” quite rapidly. Second, imitation is stronger for low frequency than for high frequency words (Goldinger, 1998; Goldinger and Azuma, 2004), for words the speaker has been exposed to several times (Goldinger, 1998; Goldinger and Azuma, 2004; but see Shockley et al., 2004), and for words that were always presented in the same voice rather than in different voices (Goldinger, 1998). This second set of results is explained in terms of the content of echoes. Less familiar words are represented by fewer traces in memory (because they are encountered less often); therefore, the contribution of individual traces (including the most recent percept) to the resulting representation is larger, and this leads to more faithful imitation of the percept in production. Conversely, the more a speaker is exposed to a particular percept and the less variability there is among instances of this percept (e.g., it is always pronounced by the same model), the stronger the influence its trace exerts on the speaker's mental representation, hence enhancing imitation.

Other studies used similar repetition tasks or speeded shadowing tasks and found evidence that participants imitated specific features of the speech signal, as indicated by objective phonetic measures. In particular, reliable imitation has been found for two acoustic dimensions: VOT (Shockley et al., 2004; Mitterer and Ernestus, 2008; Sanchez et al., 2010; Nielsen, 2011), and F0 (Bosshardt et al., 1997; Kappes et al., 2009; Babel and Bulatov, 2012). Some studies also reported imitation of speech rate (i.e., word duration; Bosshardt et al., 1997) and speech style (i.e., the use of full or reduced pronunciation variants; Kappes et al., 2009; Brouwer et al., 2010). Imitation of allophonic variants was reported by some authors (Honorof et al., 2011) but null effects were found by others (Mitterer and Ernestus, 2008).

Delvaux and Soquet (2007) showed convergence between two dialects of French. Interestingly, they automatically extracted those acoustic features that best distinguished between the two dialects (i.e., that could be most reliably used to classify utterances into dialect categories) using Discriminant Analysis; convergence was then measured as reduced distance along the dimensions thus identified. This method has the advantage that no *a priori* assumptions are made about which features are expected to be imitated.

It has been suggested that the overall impression of similarity reported by naïve listeners in AXB tasks could be realized phonetically in different ways, possibly with relatively minor adjustments along several dimensions (Pardo, 2012). This could explain why some studies have failed to confirm results from perceptual judgment tasks when they looked at a few acoustic dimensions that were hand-picked by researchers (Pardo et al., 2010, 2012).

## The motor theory of speech perception

According to the MT of speech perception, speech perception units are not defined in terms of acoustic properties of the speech signal, but in terms of articulatory gestures (Liberman and Whalen, 2000). In other words, the units of perception and the units of production are the same. Proponents of MT have also claimed that the motor system is directly involved in the perception of speech.

As noted by Galantucci et al. (2006), one prediction of MT is that imitative responses should be facilitated over non-imitative responses (i.e., speakers should produce a speech unit faster when they have just perceived the same speech unit than when they have just perceived a different speech unit). There is evidence that this is the case, irrespective of whether presentation is in the visual modality (i.e., a silent video showing mouthing) or in the auditory modality (Kerzel and Bekkering, 2000; Fowler et al., 2003; Jarick and Jones, 2008; Galantucci et al., 2009). Interestingly, there is also evidence that phonetic imitation occurs when model utterances are presented only visually (Gentilucci and Bernardis, 2007; Miller et al., 2010).

This evidence is also consistent with a related approach, Direct Realist Theory (DRT) (Fowler, 1986). DRT also claims that listeners directly perceive speech gestures in the acoustic signal and Fowler (1986) mentions imitation as one type of response which is directly afforded by speech events. Unlike MT, DRT posits that actual vocal tract actions (and not intended gestures) are the objects of perception (see Galantucci et al., 2006, p. 366, footnote 7). Therefore, DRT is in a better position than MT to account for the imitation of subtle phonetic variation. However, DRT has trouble accounting for the effects of experience and amount of exposure found in AXB tasks, as it postulates a direct relationship between perception of the current event and production of the imitative response (Fowler, 1986).

## Communication accommodation theory

According to CAT, speech convergence stems from a speaker's desire to make herself more likeable to her conversational partner (Giles and Coupland, 1991). As such, convergence is just one of the strategies the speaker can use to manage the distance between her and her interlocutor (with an alternative strategy being divergence). Crucial to this view is the focus on a variety of individual and social variables that are claimed to affect the degree of convergence, as they affect the relationship between the speaker and her interlocutor. These variables range from personality traits (e.g., Natale, 1973) to attitudes toward in-group vs. out-group members (e.g., Giles, 1973), and their effect is claimed to be further modulated in complex ways by the speaker's communicative intentions and affective goals (Giles and Coupland, 1991).

A few studies have looked at whether interaction in semi-structured or spontaneous conversations leads to speech convergence. They have generally shown that convergence does occur, but is subject to a high degree of individual variability and is also affected by several characteristics of the interlocutors and of the interaction. Natale (1973) showed that, on average, interviewees converged to the interviewer's vocal intensity, but to a greater extent when the interviewees had more need for social approval (which was measured by their tendency to report themselves as similar to established social norms). Similarly, another study reported that the degree of convergence in F0 between the talk-show host Larry King and his guests depended on the status of guests, with Larry King converging more to higher status than to lower status guests (Gregory and Webster, 1996).

More recently, Pardo and colleagues (Pardo, 2006; Pardo et al., 2010) reported speech convergence between pairs of participants conversing together to solve the Map Task (Anderson et al., 1991). In the Map Task one participant (the giver) describes a route through a map with labeled landmarks. The other participant (the receiver) has to draw the route described by the giver on a different map, which has no labels for the landmarks. The giver and receiver cannot see each other. Pardo and colleagues (Pardo, 2006; Pardo et al., 2010) asked listeners to judge how similar tokens produced by the giver were to tokens produced by the receiver (and vice versa). The tokens were either elicited before the interaction, recorded during the interaction (either early or late into the dialog), or elicited after the interaction. Dialog partners progressively converged over the course of the interaction and remained more similar after the interaction had ended. However, the degree of imitation of one's partner varied greatly depending on at least three factors: participants' gender, conversational role (i.e., whether they were givers or receivers; Pardo, 2006; Pardo et al., 2010), and intention to imitate (i.e., whether the participants had been explicitly instructed to imitate or not; Pardo et al., 2010). This set of findings is not easy to interpret, partly because of the relatively small sample sizes (6 pairs in Pardo, 2006; 12 pairs in Pardo et al., 2010).

Overall, within these samples, males appeared to converge more than females (only same-gender pairs were tested) and instruction givers tended to converge to their partner more than their partner did to them. Pardo et al. (2010) noted that the effect of gender contradicts previous findings by Namy et al. (2002), as they showed that females tended to accommodate more than males in a shadowing task. Regarding the effect of conversational role, Pardo and colleagues also suggested that it is at odds with suggestions that convergence tends to happen in the direction of the interlocutor who takes on a more dominant role during the interaction (the giver in this case). However, we note that in other collaborative dialog tasks in which one participant acts as the instruction giver and the other as the receiver, the giver similarly tends to accommodate to the receiver, for example adopting his or her perspective (Schober, 1993), particularly when the cognitive burden of the task is shifted toward the receiver (Mainwaring et al., 2003; Schober, 2009).

Interestingly, Kim et al. (2011) showed convergence between interlocutors who were engaged in a more symmetric conversation. Participants conversed to identify the differences between two depictions of the same scene. The amount of convergence was affected by the linguistic distance between the participants. Pairs of interlocutors who spoke the same dialect of American English converged more than pairs that used different dialects. Quite surprisingly, the amount of convergence in the latter group was no different from pairs in which one of the interlocutors was a non-native speaker of American English with a clearly foreign (Korean or Chinese) accent. This suggests that dialectal differences have a strong influence on speech imitation, though the study does not determine the level at which these differences operate. In particular, it is not clear whether dialectal differences affect the degree of convergence because they correlate with different (perceived) attitudes or whether they directly determine the ability of speakers to implement phonetic imitation (because of different phonetic repertoires).

A few studies have investigated the role of attitudes directly. Pardo et al. (2012) looked at long-term convergence in a small sample of college roommates, and found a marginally significant correlation between self-reported closeness and amount of convergence. In addition, Babel (2012) reported that the degree of imitation in a repetition task was affected by the participant's own gender in interaction with other factors, such as whether the model's face was visible or not and how attractive the participants rated the model to be. Finally, Babel (2010) explicitly manipulated attitudes toward the model. She asked speakers of New Zealand English to shadow a model who was a speaker of Australian English. Convergence was unaffected by whether the model was presented as having a positive, neutral, or negative attitude toward New Zealand. However, there was a positive correlation between participants' pro-Australia bias and the extent to which they accommodated to the model's speech.

Overall, CAT has correctly pointed out that social factors and variables relating to the nature of the interaction are crucial when it comes to understanding speech convergence. There is evidence that such variables affect the degree of convergence, but it is not clear which variables or constructs best predict convergence, or what are the underlying cognitive mechanisms by which interlocutors' attitudes and beliefs affect convergence of lower-level processes involved in speech perception and production.

## Simulation theory of speech perception

Forward models map from motor commands to the motor and sensory consequences of executing those motor commands. For example, if a command is sent to the *orbicularis oris* muscle (which causes a constriction of the lips), then a forward model can be run ahead of executing the command and can allow the prediction that the lips will be rounded. Such prediction could specify different kinds of information: the change in relative position of the upper and lower lip; the kinesthetic feeling associated with rounding; the acoustic consequences of rounding (e.g., lowering of formant frequencies for vowels).

According to some researchers, forward models are routinely used for the online control of one's own actions (Wolpert and Flanagan, 2001; Wolpert et al., 2003). Various authors have proposed that a similar mechanism might underline the control of articulators during speech (Guenther et al., 2006; Tian and Poeppel, 2010; Hickok, 2012). This mechanism would be responsible for sensorimotor adaptation, which is well-documented for speech (Houde and Jordan, 1998).

According to the ST of speech perception, perception of other people's speech involves covert simulation of their speech, and covert simulation is achieved by running forward models of one's own speech production system. Similar claims have been put forward for action perception in general (Wilson and Knoblich, 2005). First, a motor command is recovered using a combination of prior knowledge and perceptual input. This command constitutes the perceiver's representation of the goal underlying the observed unfolding action. Then, the perceiver derives the motor command that is most likely to follow, and feeds it into a forward model. The output of the forward model is the predicted sensory input if the motor command were executed. Predicted input can be compared to actual input (i.e., to a perception of the unfolding action) and the resulting “prediction error” can be used to adjust the motor command. This theory is related to other accounts that posit simulation as the basis of thought (Hesslow, 2002) and imagery (Hesslow, 2002; Grush, 2004). However, it differs from Hesslow's (2002) theory in that it specifically claims that simulation is supported by forward models (rather than general associative mechanisms), in the manner proposed by Grush (2004).

Here, we propose, in line with Pickering and Garrod (2007, 2013) and Adank et al. (2010), that comprehenders can covertly simulate another's speech. Pickering and Garrod (2013) proposed that comprehenders use a combination of inverse and forward models during perception of speech. Inverse models map from a perceptual representation of the speech input to the production command that the comprehender would use if he were to produce the perceived speech himself. The production command specifies the message and includes information about communicative force (e.g., interrogative), pragmatic context, and a non-linguistic situation model (for details, see Pickering and Garrod, 2013; Figure 6).

Imagine a situation where the comprehender has no prior information about the message or the particular speaker. Correctly recovering the production command is likely to be hard. However, it is nevertheless possible because there are regularities in speech and because the comprehender has had extensive previous experience with speech in his native language (so he will have at least some general expectations about how words sound, as well as some general knowledge of how other people are likely to act in a given situation).

Once the comprehender has derived this production command, he need not rely solely on the inverse model any more. Instead, he can derive the production command which is most likely to follow the recovered production command (as if he were producing the speech himself). In turn, this drives a forward production model and a forward comprehension model, which, in combination, compute a prediction of the upcoming input. Such forward models, and the associated predictions, depend on the characteristics of the comprehender's own speech production architecture. So, for example, the forward production model could compute a prediction of the movements of the articulators and the forward comprehension model could in turn predict the acoustic features of the sound produced with that particular configuration of the articulators. Importantly, such predictions are affected by the nature of the comprehender's own vocal tract, including, for example, his fundamental frequency.

Now, if a male comprehender is listening to a female speaker, predictions based uniquely on his own fundamental frequency would mismatch the input, as the female speaker will on average have a much higher F0 than the male comprehender. However, the comprehender has conversed with many female speakers in the past and he can rely on his past experience to formulate some general prediction of how the speaker will sound. But clearly, given the extent of individual variability, such predictions would still mismatch the input. When predicted and actual perceptual representations are compared, this would generate a prediction-error signal (i.e., a measure of how much the production command needs to be modified to match the actual sensory feedback). This way, better inverse models are learned and more accurate (i.e., speaker-specific) forward-model predictions can be generated at the next time stamp.

If this process makes the comprehender's forward-model predictions more “speaker-centric,” then he will tend to implement similar corrections when he produces speech, because the forward-model architecture implicated in comprehension is the same as the one implicated in speech. The outcome will thus be phonetic imitation of the speaker he has been listening to (e.g., higher F0). Because it assumes parity between comprehension and production mechanisms, ST can also explain the finding that imitative verbal responses are facilitated (i.e., faster and more accurate) than non-imitative responses (see “The Motor Theory of speech perception”).

For this account to be plausible, however, it must be the case that comprehenders can indeed predict the phonetic properties of the speech input. Most of the evidence for prediction in language comprehension concerns predictions of semantic (e.g., Altmann and Kamide, 1999; Federmeier and Kutas, 1999), syntactic (Wicha et al., 2004; Van Berkum et al., 2005; Lau et al., 2006), or phonological properties of the upcoming linguistic input (DeLong et al., 2005). However, there is also evidence that on-line comprehension mechanisms can be fine-tuned to specific speakers, both at the semantic level (Van Berkum et al., 2008) and at the phonetic level.

For example, listeners can take advantage of the fact that /æ/ is realized as [ε] before /g/ but as [æ] before /k/ in some dialects of American English, and rule out *bag* (pronounced [b ε g]) as a potential competitor when they hear the vowel in *back* (pronounced [b æ k]) (Dahan et al., 2008). In addition, Trude and Brown-Schmidt (2012) showed that individual speakers' phonetic characteristics can be accessed very rapidly. They used the same dialectal phenomenon as Dahan and colleagues, but exposed listeners to both the standard and the dialectal pronunciations realized by two different speakers (a male and a female). Speaker identity was varied on a trial-by-trial basis. Listeners' eye-movements to objects in a visual scene were guided by phonetic variation in the critical vowel within 300 ms of word onset. This was most evident when listeners could identify the speaker before hearing the critical word, using linguistic or pictorial contextual information, thus suggesting that contextual information guided listeners' expectations.

Speaker-specific adaptation of this kind does not necessarily demonstrate that speaker-specific predictions are computed, as it could reflect *post hoc*, ease-of-integration effects (Kutas et al., 2011). However, if listeners in Trude and Brown-Schmidt's study were covertly simulating the speakers' speech using forward models of their own production system, they could have adjusted such models very rapidly using prediction errors generated during the perception of the onset of the critical word, when they did not know *a priori* which speaker they were going to hear. If this was the case, we would expect these listeners to show phonetic imitation if they were asked to repeat the words produced by the speakers immediately after hearing them (Goldinger, 1998). When they knew the speaker's identity beforehand or when a richer linguistic context gave them more time to adjust their prediction before the critical word, listeners' expectations appeared to be stronger and more accurate. In other words, their forward models became more “speaker-centric,” and we would expect their productions to sound more similar to the speaker's.

Incidentally, ST predicts that listeners should be better at covertly simulating themselves than other speakers and, by extension, they should be better at covertly simulating other speakers the more they are similar to them. Interestingly, the robust McGurk effect is attenuated when the pre-recorded auditory stimulus is in one's own than in another's voice (Aruffo and Shore, 2012), suggesting that participants weight acoustic information (vs. visual information) more when listening to their own voice than other people's voices. Note that the time-course of activation in auditory areas during audio-visual speech perception is consistent with the hypothesis that visual information is used to generate predictions of yet-to-be-perceived sounds (Arnal et al., 2011).

In addition, Adank et al. (2009) found that speakers of Standard English are better at comprehending sentences spoken in Standard English than in Glaswegian English or in Spanish-accented English. However, the same study also showed that speakers of Glaswegian English were equally good at comprehending sentences spoken in Glaswegian English and in Standard English, indicating that exposure to an unfamiliar accent can improve comprehension of that accent even if it is very different from the listener's own accent (see also Bradlow and Bent, 2008). This is compatible with ST, as it predicts that experience with comprehending a particular accent should lead to adaptations in the listener's forward model. Crucially, ST predicts that perceptual adaptation should proceed in parallel with changes in production. Interestingly, Evans and Iverson (2007) reported precisely such a correlation between long-term changes in perception and production when they tested speakers of Northern English who were adapting to the standard variety spoken in the South of England. In addition, Adank et al. (2010) showed that overt imitation of an unfamiliar accent improves perception of utterances produced in that accent (under noisy conditions) more than pure exposure and repetition without the explicit instruction to imitate. The authors interpreted this as evidence that listeners who were imitating made use of simulation and could therefore better predict perceptual characteristics of the signal and filter out noise in the input.

However, it is evident that sensitivity to phonetic variation is very useful but also somewhat costly, given the high variability of phonetic realizations within individuals. Comprehenders could sometimes make use of predictions at other linguistic and non-linguistic levels to provide converging evidence for their predictions of upcoming input. By doing so, they might become less sensitive to small deviations of the actual perceptual input from the predicted perceptual input at the phonetic level (as they might be more confident in their predictions at other levels).

This could explain the finding that phonetic imitation is less pronounced for high frequency words (Goldinger, 1998; Goldinger and Azuma, 2004), under the assumption that high frequency words are also more predictable in general (Bell et al., 2009). In addition, it is consistent with Nye and Fowler's (2003) evidence that imitation occurs to a larger extent in a shadowing task when the shadowed material is further removed from the phonotactic constraints of the shadower's native language (English). In general, this predicts that phonetic imitation should be larger when there is less information at other linguistic levels on which to base predictions on (e.g., when repeating isolated words than when repeating sentences).

Interestingly, there is some evidence that the sensitivity of listeners to anomalies in the speech input varies as a function of how much (lexical) information is available. For example, mispronounced phonemes are detected less often when they are closer to the end of a word (Marslen-Wilson and Welsh, 1978) and lexical biases in the perception of ambiguous input are also stronger closer to the end of a word (Pitt and Szostak, 2012). In addition, sensitivity to subtle variations in the phonetic input can be manipulated both explicitly (i.e., by asking participants to focus on the quality of the input; Pitt and Szostak, 2012) and implicitly, with a cognitive load manipulation (e.g., Mattys and Wiget, 2011).

From the perspective of ST, the finding that listeners can be more or less sensitive to phonetic detail suggests that the extent to which prediction errors at the phonetic level are used to adjust the production command depends on the allocation of limited attentional resources. Listeners seem to favor predictions at the lexical level over predictions at the phonetic level when resources are limited. This could explain the differential effects of explicit instructions to imitate vs. unintentional imitation reported by Pardo and colleagues (Pardo, 2006; Pardo et al., 2010). However, it must be noted that lexical biases like the ones reported above could be explained within theories of perception that do not assume covert simulation (or any production involvement for that matter; see Mattys and Wiget, 2011).

But how would social factors modulate the degree of convergence? One way in which they could is by constraining the scope of predictions about the speaker. Since social variables correlate strongly with various phonetic features (e.g., Pope et al., 2007), such variables could be used to drive predictions, especially if information about these variables is available before speech begins and needs not be extracted from the speech signal itself (as is the case for gender and socio-economic status).

For example, if a comprehender has prior knowledge about the speaker's dialect, and he has had sufficient previous experience with speakers of that particular variety, he might adjust his forward model preemptively. If, however, he has not had extensive experience with that particular dialect, it might take him time to tune in (and therefore he would display less convergence overall). This suggests that evidence for the influence of attitudes on the extent of convergence (Babel, 2010) might be recast in terms of the degree of contact with a particular dialect (i.e., it is possible that more positive attitudes correlate with more extensive exposure to a particular variety).

On a more general level, however, social variables might affect listeners' tendency to rely on forward-model predictions during comprehension of a speaker's utterances. Pickering and Garrod (2013) argued that comprehension can proceed through two different routes; the prediction-by-simulation route, which makes use of forward models, and the prediction-by-association route. The latter is also used to predict perceptual events that are not produced by an intentional agent (e.g., the rustling sound of leaves on a windy day). The association route might sometimes play a stronger role than the simulation route in speech comprehension, particularly when the speaker is perceived by the listener as dissimilar to himself. Crucially, whenever comprehension proceeds preferentially through the association route, evidence for imitation of the speaker's speech should be reduced, because this route does not entail the involvement of production mechanisms in comprehension.

In fact, the simulation route could potentially fail when the distance between interlocutors is large. Listeners could learn to anticipate potential failures, and rely on the simulation route more when they perceive the speaker as being more similar to them than when they perceive the speaker as being very dissimilar. This raises the possibility that people might be more likely to imitate speakers they aspire to be similar to, which in turn would explain why high status speakers tend to attract more convergence than lower status speakers (Gregory and Webster, 1996).

To the extent that attitudes toward the speaker can influence this perception of similarity/dissimilarity, then they should affect the likelihood of the simulation route being preferred to the association route and, therefore, the degree to which phonetic imitation takes place. Note that in this case attitudes would directly influence imitation, rather than indirectly (as assumed above) through their correlation with experience. However, the two mechanisms can reinforce each other: as a listener gains more experience with a particular variety, he might develop more positive attitudes toward that variety, which, in turn, might increase the likelihood of perceiving speakers of that variety as more similar to himself, and thus triggering the use of the simulation route in comprehension.

The assumption that perceived (as well as actual) similarity triggers reliance on the simulation route over the association route is necessary to explain how large differences in speech can be overcome. Pickering and Garrod (2013) proposed that prediction-by-association will be emphasized when the comprehender is less similar to the producer (e.g., when the comprehender is a native adult speaker of the language and the producer is a non-native speaker or a child), but it is possible that social bonds might sometimes increase the perceived similarity between a native and a non-native speaker or a parent and a child and thus favor the simulation route and its consequences (including some degree of phonetic convergence).

Finally, interaction-related variables (e.g., conversational role) have been shown to affect the extent of imitation. Pickering and Garrod (2013) suggested that use of the simulation route could be primed in situations where listeners take on the role of speakers as well (i.e., in dialog) and specifically when episodes of comprehension are tightly interwoven with episodes of production (i.e., interactive unstructured dialogs vs. structured exchanges with longer turns and less feedback). The extent to which these features of the interaction affect imitation has not been investigated yet. It would be interesting to study, using a similar rationale to Adank et al. (2010), whether comprehension of an unfamiliar accent can be enhanced by engaging listeners in dialogic exchanges with speakers of that accent. We predict that the more interactive the dialog, the more listeners will imitate the accent and the better they will then become in understanding sentences spoken in the unfamiliar accent against background noise.

As for existing evidence, conversational role does seem to matter, as instruction givers converged to a greater degree than instruction receivers in Pardo's studies (Pardo, 2006; Pardo et al., 2010, 2013). We speculate this could be due to the fact that interlocutors performing different roles might have engaged in production to varying degrees over the course of the interaction. Interestingly, a recent study by Pardo and colleagues (Pardo et al., 2013) provides tentative support for this hypothesis. They used a similar paradigm to their previous studies with the Map Task, but asked participants to switch roles throughout the experiment, so that the participant who acted as giver on the first round became receiver on the second round (and giver again on the third, and so on). They reported that participants who acted as givers on the first round tended to speak for longer on all rounds, irrespective of subsequent role changes. Interestingly, only these participants showed phonetic convergence (when they were acting as receivers, as assessed in an AXB listening task), consistent with our hypothesis that convergence is enhanced when the production route is more active.

Clearly, however, interlocutors might converge very little (or even diverge from each other), while nonetheless tightly interweaving production and comprehension. As outlined above, ST assumes that the larger the distance between interlocutors (i.e., the more dissimilar they are and/or perceive each other to be), the less they will tend to rely on the simulation route and, conversely, the more they will use the association route in comprehension. On the contrary, the perception of similarity (whether accurate or not) triggers the use of simulation, which is turn leads to convergence and increased (actual, as well as perceived) similarity. This guarantees that the forward model can be used interchangeably to predict one's own and another's speech in the context of a conversation (at least to some extent, and the more so when interlocutors align on other levels as well).

Overall, ST provides explanations for at least some of the effects of social variables that have been reviewed above. Importantly, ST explains such phenomena within a mechanistic framework. Intentions and attitudes can affect imitation by assigning more weight to the simulation or the association route during speech comprehension. In addition, as we have argued, many of the findings traditionally interpreted as intention-driven (within the context of CAT, where convergence is a conversational strategy) could in fact emerge from the dynamics of interaction. It is possible that imitation may sometimes serve as an intentional signal with an intrinsic communicative value (e.g., “I want you to like me”; cf. Pardo, 2012), but we propose that it generally occurs as a by-product of the internal mechanics of speech comprehension.

## Discussion

The review of the literature on speech imitation and convergence revealed that a large proportion of studies relied on naïve listeners' subjective judgments to establish whether imitation occurred, but many studies have also looked at measurable properties of the speech signal. Few studies compared listeners' judgments with phonetic measures and they found little support for a direct mapping between judgments and phonetic measures (Pardo et al., 2010, 2012). For the most part, evidence that specific features of the speech signal are imitated is scant, with contradictory findings across studies and also considerable individual variation within studies in many cases. Nevertheless, the impression of increased similarity after exposure to a model, as reflected in listeners' judgments, has been replicated several times, both in laboratory tasks (e.g., Goldinger, 1998) and in more naturalistic settings (e.g., Pardo, 2006). This suggests that speech imitation is a reliable phenomenon, but its objective correlates are yet to be identified.

Abstracting from the reliability and directionality of single findings, data are clearly consistent with two assertions: (i) that speech perception influences speech production; (ii) that the link between speech perception and speech production is mediated by a number of variables (Pardo, 2006, 2012; Pardo et al., 2010). The first assertion (i) is incorporated in three of the four theories we have presented: the ET, the MT, and the ST. Specifically, the three theories all posit some form of parity between perception and production in order to explain the fact that one influences the other. Incidentally, a very similar line of reasoning has been applied to perception and production of actions other than speech, where evidence of cross-influences between action and action perception (Prinz, 1997) has been taken to support the existence of a shared representational code (Hommel et al., 2001).

Additionally, MT makes the further assumption that this common code uses the “vocabulary” normally thought to underlie speech production, rather than the one which has been postulated in acoustic theories of speech perception. It is important to note that this claim is not shared by ST, notwithstanding its emphasis on forward production models. In fact, ST claims that both production and comprehension processes (and representations) are involved in comprehension as well as in production.

The second assertion (ii) is compatible with all theories except MT, which assumes that the link between perception and production is direct and unmediated. However, ET can easily accommodate only a subset of the variables which have been shown to mediate imitation effects: those that have an effect on the content and strength of memory traces (e.g., amount and consistency of input). CAT, on the other hand, seems to allow for an almost unlimited inventory of variables to mediate imitation (Gallois et al., 2005), but it is short of explanations as to how such variables can interact with the processes of perception and production to bring about the observed effects. As a result of this, we argue, CAT makes also very few clear, specific, and testable predictions.

On the contrary, we argue, ST is very explicit about mechanisms. In a nutshell, it proposes that the mechanisms underlying imitation are similar to the mechanisms underlying sensorimotor adaptation. We have also shown how it could account for some of the mediating variables that research has identified and shown that its explanatory scope is potentially much wider than that of ET. Below, we first discuss the extent to which existing evidence supports the assumptions of ST about the mechanism involved in speech perception. Then, we briefly review a few novel and testable claims that follow from ST's account of speech imitation.

As discussed in Section “Simulation Theory of Speech Perception,” there is substantial evidence for prediction in language comprehension, at the levels of semantics, syntax, and phonology. In addition, there is some indication that prediction in language comprehension uses production processes, with some evidence relating to the phonological level (see Pickering and Garrod, 2013). D'Ausilio et al. (2011) repeatedly exposed participants to a pseudo-word (e.g., *birro)* and used TMS to reveal immediate appropriate articulatory activation (associated with *rr*) when they heard the first part of the same item (*bi*, when coarticulated with *rro*) compared to when they heard the first part of a different item (*bi*, when coarticulated with *ffo).* However, it is possible that such activation is incidental and therefore we cannot be certain that activation of production processes plays a causal role in prediction. Therefore, more studies are needed before it can be safely concluded that predictive processes in perception are production-based.

In addition, we are not aware of any study that specifically investigated whether predictions can occur at the phonetic level in speech perception. While the findings of Trude and Brown-Schmidt (2012) indicate that listeners can integrate speaker-specific phonetic information very rapidly to guide on-line comprehension, they do not demonstrate that phonetic predictions are computed (i.e., that specific phonetic features of the upcoming input can be anticipated by listeners). Finally, Adank et al.'s (2010) findings indicate that overt imitation of phonetic features enhances perception, but they do not directly show that this occurs because of the effects of imitation on prediction, such as assumed by ST. In conclusion, evidence for ST is mostly indirect at this stage.

Nevertheless, this theoretical framework is appealing because it has the potential to explain a wide range of findings about speech perception, production and, most importantly for our current purposes, speech imitation. The appeal of ST, especially in comparison with other accounts of speech imitation, is that it accommodates many of the existing findings while also making new claims (see Section “Simulation Theory of Speech Perception”). For example, ST predicts that phonetic imitation should be greater for isolated words than words in context and for words that are less predictable given the preceding context than for words that are more predictable.

In addition, ST predicts that phonetic imitation should be greater the more the simulation route is used in perception. Use of the simulation route should be enhanced, according to the theory, in two ways. The first factor is the level of activation of production processes at the time of perception: the more a listener is engaged in production (e.g., as a result of taking turns with an interlocutor), the more he will use the simulation route in perception. Pardo et al.'s (2013) finding that interlocutors who took a leading role at the start of the interaction imitated more than interlocutors who did not is consistent with this claim, but future studies should directly investigate this hypothesis.

The second factor is the (perceived and actual) similarity between the listener and the speaker: the more the listener is similar to the speaker, and also the more the listener perceives himself as similar to the speaker, the more he will use the simulation route in perception (as opposed to the association route). This claim could be tested by having speakers imitate two models, one whom they perceive as more similar to themselves, and one whom they perceive as less similar to themselves. Overall subjective similarity could be measured with questionnaire ratings. Perceived similarity at the phonetic level could be measured with an AXB task.

In conclusion, we would like to suggest that research in the domain of speech imitation could benefit from the new insights brought about by the ST of speech perception when it comes to reasoning about the relationship between speech perception and production.

### Conflict of interest statement

The authors declare that the research was conducted in the absence of any commercial or financial relationships that could be construed as a potential conflict of interest.
